# The psycholinguistic and affective structure of words conveying pain

**DOI:** 10.1371/journal.pone.0199658

**Published:** 2018-06-29

**Authors:** Eleonora Borelli, Davide Crepaldi, Carlo Adolfo Porro, Cristina Cacciari

**Affiliations:** 1 Department of Biomedical, Metabolic and Neural Sciences, University of Modena and Reggio Emilia, Modena, Italy; 2 Center for Neuroscience and Neurotechnology, University of Modena and Reggio Emilia, Modena, Italy; 3 Department of Cognitive Neuroscience, International School for Advanced Studies (SISSA), Trieste, Italy; 4 Milan Center for Neuroscience (NeuroMi), Department of Neurology and Neuroscience, University of Milano-Bicocca, Milan, Italy; University of Rome, ITALY

## Abstract

Despite the flourishing research on the relationships between affect and language, the characteristics of pain-related words, a specific type of negative words, have never been systematically investigated from a psycholinguistic and emotional perspective, despite their psychological relevance. This study offers psycholinguistic, affective, and pain-related norms for words expressing physical and social pain. This may provide a useful tool for the selection of stimulus materials in future studies on negative emotions and/or pain. We explored the relationships between psycholinguistic, affective, and pain-related properties of 512 Italian words (nouns, adjectives, and verbs) conveying physical and social pain by asking 1020 Italian participants to provide ratings of Familiarity, Age of Acquisition, Imageability, Concreteness, Context Availability, Valence, Arousal, Pain-Relatedness, Intensity, and Unpleasantness. We also collected data concerning Length, Written Frequency (Subtlex-IT), N-Size, Orthographic Levenshtein Distance 20, Neighbor Mean Frequency, and Neighbor Maximum Frequency of each word. Interestingly, the words expressing social pain were rated as more negative, arousing, pain-related, and conveying more intense and unpleasant experiences than the words conveying physical pain.

## Introduction

May words be painful? Undoubtedly yes and in several respects, as literary sources, personal experience, and a handful of recent behavioral and brain-imaging studies have shown (e.g., [1–3]). Words represent the main tool for describing the physical and social experience of pain (e.g., [4–5]) and can be metaphorically extended to characterize social phenomena, as exemplified by the title of a recent article in *Science*: “Growing pains for global monitoring of societal events” [6].

Notwithstanding the pervasiveness and relevance of the words used to convey pain at different levels (henceforth pain words), the psycholinguistic and affective characteristics of this important part of the lexico-semantic domain of negative words have never been specifically tested. Norms about affectively-laden words already exist for a variety of languages, including Italian (e.g., [7–8]), but due to the general aim of these datasets they contain only a limited number of pain words (we return on this point below). This study was devised to bridge this gap creating a normed corpus of Italian pain-related words (Words of Pain database, henceforth WOP). WOP may at the same time contribute to the literature on the characteristics of affectively-laden words and provide a tool for experimental studies of pain.

Language is more than a mere medium when it comes to share our pain experiences. In fact, it has been shown that processing pain-related words is associated with enhanced activation of part of the neural circuitry underlying physical pain experiences [1,2,9,10]. Medical studies also have observed that the presentation of pain words can modulate the perception of noxious stimuli, especially in chronic pain patients [3]. The mechanisms underlying these important effects of pain words are still under investigation. It has been suggested that the comprehension of pain words may occur via an embodied simulation involving reliving and/or retrieving pain-related information (e.g. [11]), in analogy to what happens in the empathic response to pain (e.g., [12]). In fact, merely observing, thinking about, or inferring that someone else is in pain have been shown to trigger the emergence of physical pain [13], a phenomenon known as *synesthesia* for pain [14–16]. A wealth of studies on empathy for pain has led to suggest the existence of common neural substrates that map the perception of pain in oneself and in the others (for an overview, see [13]).

### Describing pain in medical settings

We use linguistic stimuli to convey our own experience of pain since early childhood [17]. From a medical viewpoint, assessing the sensory, affective, and cognitive impact of the pain experience to the sufferers still represents a challenge [18,19]. “Pain is defined and ultimately evaluated by subjective report. Much can be inferred from objective measures of anatomy, physiology, and behavior, but verbal report remains the standard by which all other measures are compared” ([19], p.1309). In fact, medical doctors typically categorize the pain of sufferers primarily “translating” their pain reports into a finite set of descriptors that are thought to “capture and categorize facets of the pain experience as evidenced in the endorsement and ranking of pain descriptors” ([19], p. 1387). These descriptors are contained in pain questionnaires devised to assess different types of pain. For instance, in one of the sections of the *McGill Pain Questionnaire* (MPQ, [20]; for an overview see [18,19]), the pain sufferer is asked to indicate what his/her present pain feels like choosing among 78 descriptors (e.g., *fearful*, *itching*, *hot-burning*). According to Melzack (1975), these pain descriptors reflect three distinct components of pain that be divided in “sensory descriptors” that convey the sensory qualities of pain (e.g., *burning*), “affective descriptors” that convey the emotional components of pain (e.g., *punishing*), and “evaluative descriptors” that provide a global evaluation of the pain experience (e.g., *unbearable*). However, since the MPQ was primarily designed by clinical doctors (as all the other pain questionnaires), the verbal items were not controlled for any of the psycholinguistic and emotional variables that are known to modulate the cognitive demands of their processing.

### Pain words and the affective lexicon

Pain words are part of the general domain of affectively-laden words. Consensus exists about the fact that the affective space is best characterized by a two-dimensional structure formed by two orthogonal dimensions that together account for most of the variation in how affective stimuli are evaluated [21–23]. Valence ranges from positive to neutral to negative and is thought to reflect the general motivational significance of a stimulus. Arousal ranges from low to high and is thought to reflect the degree to which a stimulus prepares a person for action or captures and focus attention [21,24]. Most current models of affective word processing assume that valence and arousal are orthogonal variables ([25,26]; for an overview of consistent and inconsistent results, see [27–29]).

In general, affectively-laden words (and sentences) are processed faster and more efficiently, elicit larger electrophysiological responses since very early processing stages and activate affect-related brain regions (e.g., medial PFC, ACC, insula, and amygdala) more strongly than affectively-neutral linguistic stimuli (for overviews, see [30–32]). That affective connotations facilitate processing may reflect the grounding of these word meanings in bodily emotional experiences [33,34].

A wealth of studies has shown that negatively valenced information is associated with more complex mental representations that require a more demanding cognitive processing than positively valenced information (*Negativity bias*, [35,36]). Unpleasant events or stimuli, compared to matched pleasant ones, evoke larger emotional responses, longer duration responses with a broader impact on the cognitive system. According to the *Automatic Vigilance Hypothesis*, humans preferentially attend to negative stimuli and this attention to negative Valence diverts processing resources away from other stimulus properties, leading to longer response times [37–42]. Indeed, negative words typically elicit slower color naming [43], lexical decisions ([39–44] but see [45] for the mitigating role of arousal), and word naming [46] than neutral and/or positive words. This would reflect the fact that survival primarily depends on our ability to withdrawing from negative events and scenario [47]. Since the withdrawal-aversive system has a processing priority over the approach-appetitive system [48], negative stimuli recruit more attentional resources than positive stimuli. This hypothesis has been supported by word studies using different tasks [46,49–51]. However, recent experiments have questioned this negative emotion processing advantage showing that once the non-emotional characteristics of words (e.g., length, frequency, and orthographic neighborhood) were considered, and neutral control words were used as well, much of the processing difference between negative and positive words disappeared ([44] but see [52]). In some cases, the asymmetry was even reversed with a processing advantage for both positive and negative words over neutral words [29,47,53]. Then, in an ERP study, Hofmann et al. [45], showed that lexical decision responses were speeded at a similar extent for positive and high-arousal negative words suggesting that the level of arousal differently interacts with positive and negative valences in early lexical processing.

### Physical pain and social pain

According to the International Association for the Study of Pain (IASP), physical pain is defined as the unpleasant sensory and emotional experience associated with actual or potential tissue damage or described in terms of such damage. Physical pain is often associated with a noxious physical stimulus. However, painful experiences are triggered not only by noxious stimuli but also by events, feelings, and thoughts that usually lead individuals to experience a form of pain that recently has been defined as social pain [5,54] (although it incorporates also aspects of a more general feeling of pain not necessarily associated to social events). Social pain is thought to derive from social exclusion, rejection, loss and grief (e.g., [55,56]) and generally is described as intense as actual, physical pain [57].

Across languages we extend the use of physical pain words to describe experiences of social pain (e.g., *broken heart*, *soul scar*) (e.g., [5,58]). This use can be epitomized by the words of Hillary Clinton in her first speech after 2016 US election defeat, “This is very painful and will be for a long time” [59]. There is now growing consensus that the use of physical pain words to describe social pain is more than just a convenient metaphor. In fact, several brain-imaging studies have shown that the painful feelings following social pain rely on some of the same neural regions sub serving physical pain processing (e.g., [54,55], but see [60,61]). Notwithstanding the fact that social pain is mostly expressed using physical pain words, the stimuli of many behavioral and brain-imaging studies on social pain were not words but rather other type of visual stimuli (e.g., pictures, the Cyberball paradigm; for overviews see [60,61]).

### Why creating word corpora?

Many studies investigating human cognition use tasks that require verbal stimuli as experimental material because words can be tightly controlled for their attributes [62]. Therefore, using stimuli controlled for the psycholinguistic and affective variables that are known to affect the time it takes to encode a word has become crucial. This has led to the growth of large-scale studies in different languages aimed at creating databases providing normative information about the most important variables affecting lexico-semantic processing (e.g., *English Lexicon Project*, [63]; *French Lexicon Project*, [64]; *Dutch Lexicon Project*, [65]). Typically, these normative data are obtained from rating and/or reaction times studies in which participants evaluate these variables and/or perform word recognition tasks. These large-scale studies produce databases offering psycholinguistic, affective, and behavioral measures rated by large numbers of participants (e.g., [66–69]). Other databases provide normative data about specific set of words or specific psycholinguistic, semantic, and/or affective characteristics of the stimuli (e.g., affective words [70,71,7,8]; nouns [72]; monosyllabic words [73]; idiomatic expressions [74]; semantic categories [75,76]). Italian databases providing psycholinguistic, semantic, and/or general affective normative about sets of Italian words are available as well (e.g., [7,8,76–84]). However, none of them is specifically focused on pain words, nor they include a number of pain-related items to make them suitable for pain experiments.

For many years, research on emotion has predominantly used the *Affective Norms for English Words (ANEW*, [85]). ANEW provides a set of normative data about the valence, arousal, and dominance of 1,034 American English words. Language-specific adaptations of the ANEW are now available for many languages including Italian [7,8], Brazilian Portuguese [86], Chinese [87], Dutch [70,71,88], European Portuguese [89], Finnish [90], French [91], and Spanish [92]. Other datasets on affective words have been proposed (e.g., [69,93–95]), some of which also provide ratings of lexico-semantic variables and/or lexical decision times for larger set of stimuli (e.g., [96]). Concerning Italian, Montefinese et al. [7] and Fairfield et al. [8] collected ratings for psycholinguistic and affective variables of 1,121 Italian words (extending the original ANEW) respectively from younger and older adults. Due to the general aims of these databases, only a few of the words we use to convey pain were included. For instance, the 1121 words tested in Montefinese et al [7] only included 76 of the pain words of WOP. More importantly, WOP differs from these databases in that it offers not only the psycholinguistic and affective characteristics of 512 words, but also ratings related to pain-related variables (see below) relevant to the research on pain.

### The present study

In this study, we selected 512 Italian pain words including (1) nouns referring to objects, conditions, events, and feeling that may cause physical pain (e.g., *ago*, *needle*; *malattia*, *illness*) or social pain (e.g., *abbandono*, *abandon*; *lutto*, *grief*); (2) adjectives that describe physical or social pain (e.g., *atroce*, *dreadful*), painful objects (e.g., *appuntito*, *pointed*), and painful events and moods (e.g., *deprimente*, *depressing; inconsolabile*, *inconsolable*) and adjectives that convey sensory as well as emotional aspects of pain (e.g., *addominale*, *abdominal*; *diffuso*, *radiating* as well as *costrittivo*, *constrictive*; *fastidioso*, *uncomfortable*); (3) verbs referring to pain, painful objects, and actions that may be painful or cause pain (e.g., *bruciare*, *to burn; sbattere*, *to stab*). For each of these words, we collected ratings concerning psycholinguistic (Familiarity, Age of Acquisition, Imageability, Concreteness, Context Availability) and affective properties (Valence, Arousal). We also tested how much each of these 512 words is associated to pain (Pain-relatedness) and how intense and unpleasant is the pain experience conveyed by their meaning (Pain Intensity and Pain Unpleasantness, respectively). According to the experimental literature on pain, Intensity taps on the sensory-discriminative dimension of pain (i.e., the physical characteristics of the noxious stimulus, namely how intense is the pain) and Unpleasantness taps on the affective-motivational dimensions of pain (i.e., its emotional characteristics, namely how much disturbing is the pain) [97]. In addition, we collected data concerning the Length, Written Frequency, N-Size, Orthographic Levenshtein Distance 20, Neighbor Mean Frequency, and Neighbor Maximum Frequency of each word.

We also analyzed the three word classes (i.e., nouns, adjectives and verbs) separately since there is evidence that word class affects the timing and characteristics of affective word processing (e.g., [32,98–100]. This could reflect the fact that, as Palazova et al. [100] pointed out, adjectives that typically describe characteristics, states, and traits may have a more direct link with emotions than verbs, that typically describe actions or events, and then nouns, that denote more or less concrete objects. Finally, we analyzed the psycholinguistic, affective and pain-related differences between physical and social pain words.

## Materials and methods

### Participants

1020 undergraduates, PhD students, postdocs, and senior researchers (276 male and 744 female; age range: 18–40, mean age: 24.2 years, SD = 4.3) of the Universities of Parma, Modena and Reggio Emilia volunteered to participate in this online study. They were all Italian native speakers. Participants were recruited through an e-mail sent to the specific mailing lists of these Universities. The study was performed in accordance with the ethical standards of the 2013 Declaration of Helsinki and was approved by the Departmental Ethics Committee of the International Advanced Studies Institute, SISSA.

### Materials

The stimulus set consisted of 512 Italian words associated to pain. To select the words, we used an extraction procedure typical of the computational linguistic research. This procedure assumes that the lexicon is a metrical space in which words are separated by distances that depend on the degree of semantic similarity between words measured through their statistical co-occurrence distribution in texts [101]. We used the word *dolore* (*pain*) as an anchor point and selected the content words co-occurring with it in a window of 25 words to the left and 25 words to the right of *dolore* in a corpus of Italian newspapers’ texts (*La Repubblica Corpus*, [78]) as well as medical dictionaries, blogs, and pain questionnaires. The resulting word list was formed by: a) 199 nouns (in their singular form), 46 of which referred to social pain; b) 218 adjectives (in the singular masculine form), 15 of which referred to social pain; c) 75 verbs (in the infinite form), nine of which referred to social pain; d) 20 words that may belong to different classes depending on context (e.g., *cieco*, *blind; estremo*, *extreme*, *can either be nouns or adjectives*), one of which referred to social pain (e.g., *intimo*, *intimate*).

Since 48 out of the 512 words could be used to refer to both physical and social pain (e.g., *aborto*, *abortion*; *commozione*, *sentiment/concussion*), we asked 67 different participants (24 male and 43 female; age range: 19–40, mean age: 33 years, SD = 5.1) to decide whether each of these 48 words predominantly referred to physical or social pain. The percentages of choice are listed in the database. The database resulting from this selection procedure contains a lower number of words referring to social pain than to physical pain. This may reflect the fact that many of the words referring to physical pain are metaphorically extended to convey social pain as well.

### Tested variables

We tested the following variables:

(1) *Familiarity*, i.e., the frequency with which a word occurs in everyday life [102]. The rating scale went from one (*not at all familiar)* to seven (*extremely familiar*);
(2) *Age of Acquisition (AoA)*, i.e., the age at which a word was learnt [103]. The rating scale went from one (*0–2 years*) to seven (*13 and older*) with intervening points spanning two years [104]. It has been shown that AoA represents a reasonable estimate of the actual age at which a word is acquired. In fact, AoA ratings significantly correlate with more objective measures of word acquisition age (e.g., [105–108]);
(3) *Imageability*, i.e., the ease with which a word gives rise to a mental image [109,110]. The rating scale went from one (*not at all imaginable*) to seven (*extremely imaginable*);
(4) *Concreteness*, i.e., the degree to which a word refers to a perceptible entity [111,112]. The rating scale went from one (*not at all concrete*) to seven (*extremely concrete*);
(5) *Context Availability*, i.e., the ease with which a word may call to mind a context or circumstance [113]. The rating scale went from one (*context not at all available*) to seven (*context extremely available*). Although we may be more able to call to mind a context for familiar than for unfamiliar words, it has been shown that Context Availability and Familiarity tap on different aspects of language processing [114];
(6) *Valence*, i.e., the degree to which a stimulus is perceived as emotionally negative or positive [22]. The rating scale went from -3 (*extremely negative*) to +3 (*extremely positive*) through 0 (*neither negative nor positive*) [70,71] to keep a more intuitive negative to positive scale [115];
(7) *Arousal*, i.e., the excitation potential of a stimulus regardless of whether it is positive or negative [116]. The rating scale went from one (*not at all arousing*) to seven (*extremely arousing*);
(8) *Pain*-r*elatedness*, i.e., the extent to which the word was associated to pain. The rating scale went from one (*not at all associated*) to seven (*extremely associated*);
(9) *Pain Intensity*, i.e., the intensity of the pain conveyed by the word meaning. This variable was rated using a Visual Analogue Scales (VAS) [117], in analogy to the way in which it is measured in the experimental pain literature; the VAS consisted of a line of 10 cm with extremes labeled as *Not at all intense* and *Extremely intense*;
(10) *Pain Unpleasantness*, i.e., the unpleasantness of the pain conveyed by the word meaning. As per Pain Intensity, this variable was rated using a Visual Analogue Scales (VAS) [117], in analogy to the way in which it is measured in the experimental pain literature; the VAS consisted of a line of 10 cm with extremes labeled as *Not at all unpleasant* and *Extremely unpleasant*.

When the meaning of a word was unknown, subjects were instructed to choose the option "*I don’t know this word*".

Familiarity was always rated first since past research has shown that having previously seen a word could affect Familiarity ratings [118]. The variables were presented in the same order in all the questionnaires.

In addition, we collected the following data:

(11) *Word Length*, measured as number of letters;
(12) *Word frequency (Zipf)*, according to the *Subtlex-IT corpus* [80], a database of Italian word frequencies based on 130 million words extracted from film and television subtitles;
(13) *Neighborhood Size* (Nsize), namely, the number of words of the same length differing from the target word by exactly one letter [119];
(14) *Orthographic Levenshtein Distance 20* (OLD20), namely, the mean edit distance to the 20 closest neighbors. We collected this measure since Yarkoni et al. [120] identified it as a better indicator of lexical density than the Nsize;
(15) *Neighbor Max Frequency*, namely, the frequency of the most frequent orthographic neighbor, according to the Subtlex-IT corpus [80];
(16) *Neighbor Mean Frequency*, namely, the mean frequency of the orthographic neighbors, according to the Subtlex-IT corpus [80].

### Procedure

Participants received an e-mail asking whether they were willing to participate in a web survey. The e-mail also contained instructions on how to access a randomly assigned, self-paced questionnaire via a web site. The 512 stimuli were randomly distributed over twenty Google Form questionnaires each composed by 24 to 26 words (Table 1). Each questionnaire started with an introduction that explained that the aim of the study was to collect information about the words we use to describe pain in its broadest sense and specified the time approximately necessary to complete the questionnaire (45 minutes). Then the questionnaire contained questions concerning demographic information (i.e., gender, age, mother tongue, and education), and whether the responder suffered or had ever suffered of any forms of chronic pain or intense and repeated migraines. To reduce unpredictable effects of random word orders, the same word list was repeated for each of the ten variables of interest. Written instructions were presented at the beginning of each rating scale. They contained a definition of the variable to be rated, an explanation on how to use the Likert (or VAS) scale, and two examples of words rated with extreme values. The original Italian instructions and their English translation can be found in S1 Text.

**Table 1:** Descriptive analyses of each questionnaire’s sample.

			AGE		GENDER	
ID	Number of stimuli	Number of responders	M	SD	Percentage of Males	Percentage of Females
1	26	85	24.4	4.2	23.5	76.5
2	26	94	24.5	4.5	31.9	68.1
3	26	65	24.8	5.8	27.7	72.3
4	26	59	24.2	4.3	30.5	69.5
5	26	52	22	2.8	40.4	59.6
6	26	59	23.5	3.1	23.7	64.4
7	26	45	24	4.3	35.6	64.4
8	26	31	23.5	3.5	19.4	80.6
9	25	50	23.3	3.9	34	66
10	26	65	24.8	4.5	38.5	61.5
11	25	34	25.1	5.5	17.6	82.4
12	26	32	24.4	4.4	12.5	87.5
13	24	52	23.7	3.7	26.9	73.1
14	26	36	24.3	5.2	13.9	86.1
15	25	38	24.2	3.7	28.9	71.7
16	25	58	24.5	5	22.4	77.6
17	26	36	25	3.9	30.6	69.4
18	26	45	23.6	4.2	15.6	84.4
19	25	39	24.6	4	23.1	76.9
20	25	45	25.7	5.7	17.8	82.2

### Open access policy

The WOP database, in an Excel format including both raw and standardized data, is available on the web at https://figshare.com/s/188257a8c7de933ba28a.

Statistical analyses were carried out using R 3.4.0 [121] and IBM SPSS Statistics 24.0 [122].

## Results and discussion

**Fig 1:**
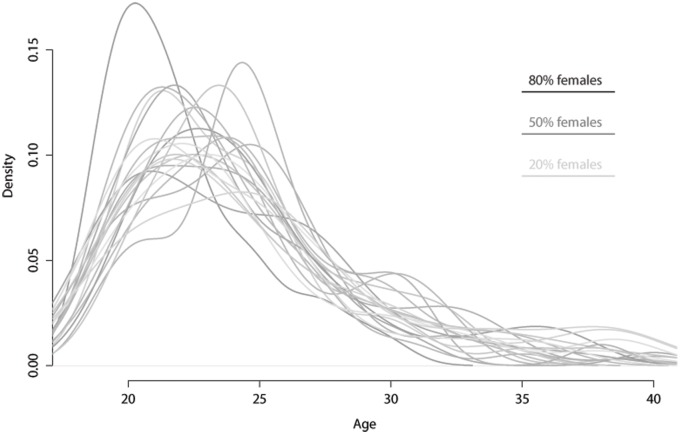
Demographic characteristics of participants. Age distribution across the 20 questionnaires, each represented by a different line. The grey scale for each line represents the gender proportion in the specific sample of participants.

Analyses of the demographic characteristics of participants (Fig 1 and Table 2) showed no significant differences in the gender of the responders to the twenty questionnaires [F (1,19) = 1.553, p = .061, η2 = .029]. A significant difference instead emerged in the mean age of the responders [F (1,19) = 1.858, p = .014, η2 = .034]. Specifically, Tukey’s HSD post hoc test revealed a significant age difference of the responders to questionnaires 5 and 20 (M = 22, SD = 2.8 and M = 25.7, SD = 5.7, respectively).

**Table 2:** Descriptive statistics for the variables considered in this study.

ALL WORDS															
	N						Mean		SD		Min			Max	
Familiarity	511						5.03		1.03		2.04			6.91	
Age of Acquisition	511						4.66		1.38		1.58			6.96	
Imageability	512						4.86		1.24		1.98			7.00	
Concreteness	512						4.41		1.39		1.63			7.00	
Context Availability	512						4.97		0.90		2.58			6.84	
Valence	510						-1.21		0.96		-2.97			2.52	
Arousal	512						4.28		1.08		1.55			6.63	
Pain-relatedness	512						4.34		1.38		1.16			6.83	
Intensity	512						54.13		20.92		4.11			96.33	
Unpleasantness	512						59.77		20.90		8.76			98.27	
	NOUNS					ADJECTIVES					VERBS				
	N	Mean	SD	Min	Max	N	Mean	SD	Min	Max	N	Mean	SD	Min	Max
Familiarity	199	5.02	0.98	2.05	6.73	217	4.93	1.09	2.04	6.91	75	5.11	0.91	2.64	6.78
Age of Acquisition	198	4.65	1.38	1.64	6.91	218	4.92	1.30	1.58	6.96	75	4.13	1.46	1.82	6.70
Imageability	199	5.42	1.01	2.74	7.00	218	4.09	1.06	1.98	7.00	75	5.64	1.10	3.07	6.92
Concreteness	199	5.19	1.22	1.89	7.00	218	3.49	1.03	1.63	6.41	75	5.11	1.06	2.48	6.80
Context Availability	199	5.31	0.77	3.11	6.64	218	4.51	0.83	2.58	6.31	75	5.30	0.86	2.61	6.54
Valence	198	-1.58	0.73	-2.93	0.19	217	-0.85	1.00	-2.93	2.52	75	-1.54	0.89	-2.97	1.05
Arousal	199	4.41	0.93	1.76	6.63	218	3.98	1.14	1.55	6.35	75	4.94	0.92	2.48	6.56
Pain-relatedness	199	4.85	1.15	2.03	6.83	218	3.70	1.35	1.16	6.83	75	5.02	1.17	2.40	6.82
Intensity	199	60.88	17.50	22.27	95.28	218	45.45	20.94	4.11	92.04	75	64.05	18.85	23.21	96.33
Unpleasantness	199	66.49	16.87	25.13	98.18	218	51.65	21.89	8.76	94.71	75	68.12	18.72	19.23	98.27
	PHYSICAL PAIN WORDS								SOCIAL PAIN WORDS						
	N	Mean	SD	Min		Max			N	Mean	SD	Min		Max	
Familiarity	439	5.03	1.04	2.04		6.91			72	5.00	0.94	2.55		6.52	
Age of Acquisition	439	4.64	1.42	1.58		6.96			72	4.78	1.18	1.73		6.79	
Imageability	440	4.90	1.27	1.98		7.00			72	4.60	0.99	2.74		7.00	
Concreteness	440	4.52	1.42	1.63		7.00			72	3.73	0.91	1.89		6.83	
Context Availability	440	4.96	0.90	2.58		6.84			72	5.02	0.85	2.61		6.44	
Valence	438	-1.09	0.95	-2.97		2.52			72	-1.96	0.61	-2.89		-0.43	
Arousal	440	4.25	1.11	1.55		6.63			72	4.44	0.90	2.65		6.50	
Pain-relatedness	440	4.27	1.41	1.16		6.83			72	4.72	1.15	2.03		6.63	
Intensity	440	52.89	21.27	4.11		96.33			72	61.67	16.97	22.27		93.53	
Unpleasantness	440	57.75	20.95	8.76		98.27			72	72.12	15.75	25.70		96.12	

Scores were standardized within subjects using a z-transformation. Because score mean and variance changed substantially across participants, and because each participant only received a subset of the stimuli, this metric was necessary for directly comparing the ratings between subjects.

Missing responses/omissions were 1.68% of the dataset. Most of these missing responses (94.63%) came from participants who reported that they did not know a given word. Unknown words could be due to the presence of a few stimuli belonging to the medical jargon (e.g., *urente*, *burning; cefalico*, *cephalic*). The mean percentage of response “*I don’t know the word*” was similar across the different variables suggesting that, in general, when participants did not know a word, they did not rate it further. Occasionally participants were able to rate only some of the variables (notably Familiarity and AoA) for words they have heard but whose exact meaning they were not sure about. The overall number of valid data points after excluding missing responses/omissions was 257,518.

Data were cleaned of uninformative/misleading data points in two steps. First, for the variables rated on 7–point scales, we excluded data points coming from participants who showed little or no variance in their responses since they had always used only one or two values of the rating scale. This procedure was applied separately for each variable and led to the exclusion of 2.58% of the data points (ranging from 0.4% for AoA to 8.8% for Familiarity). Similarly, we controlled if participants had zero variance in the Intensity and Unpleasantness ratings, meaning that likely they did not rate the words at all, leaving the cursor in the starting position. This led to the exclusion of the ratings of two participants for the Intensity scale (.19% of the available valid data points) and 11 participants for the Unpleasantness scale (1.09% of the available valid data points).

**Fig 2:**
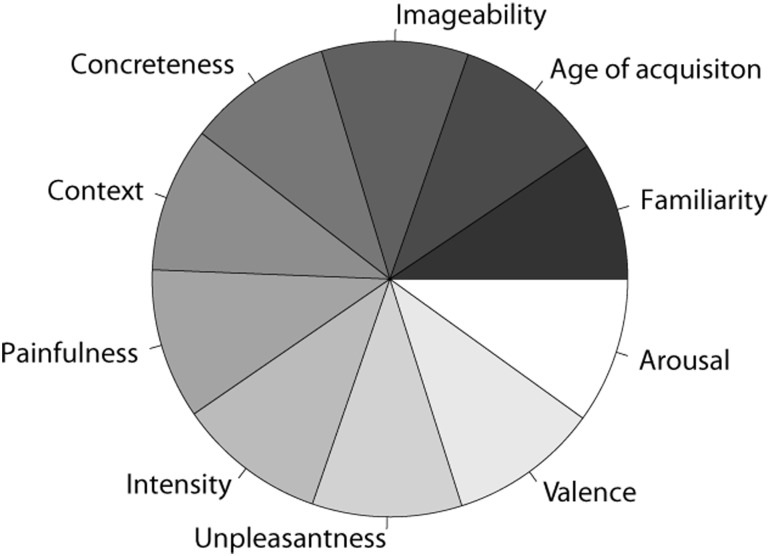
Distribution of valid data points. Distribution of the final number of valid data points (243,824) across the 10 variables of interest.

The second step allowed identifying outliers through the procedure illustrated in Rodriguez and Laio [123]. According to this procedure, participants are modeled as points in an N–dimensional space, where N equals the number of words that each participant rated. The ratings for each word define the position of each participant/point in this space, so that participants with similar judgments will be close and participants with different judgments will be relatively far apart (see data in S1 Fig). Rodriguez and Laio’s procedure was applied separately for each questionnaire and variable and led to the further exclusion of 2.72% of the remaining data points overall (ranging from .94% for Context Availability to 3.98% for Intensity). The final number of valid data points at this stage was 243,824, evenly distributed across the 10 variables of interest (Fig 2). Table 2 provides descriptive statistics of the final dataset.

We also compared the ratings obtained in the present study with those of the study on the affective lexicon of Montefinese et al. [7] for the 76 words and the variables shared by the two datasets (i.e., Familiarity, Imageability, Concreteness, Valence, Arousal). All correlations were significant (Table 3). This further suggests that our norms can be confidently used for word selection in affective word studies.

**Table 3:** Pearson’s correlations.

		WOP				
		Familiarity	Imageability	Concreteness	Valence	Arousal
Italian ANEW	R	.604**	.711**	.792**	.867**	.524**

**p < .01.

Table 2 contains untransformed values for all the words together, as well as separately for each word class, and for physical and social pain.

### Reliability of the measures

**Fig 3:**
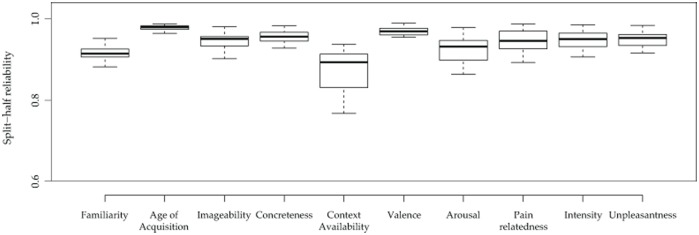
Measures reliability. Distribution Boxplots of the overall half-split reliability distributions over 1,000 random replicates, run separately for each questionnaire and for each variable.

**Fig 4:**
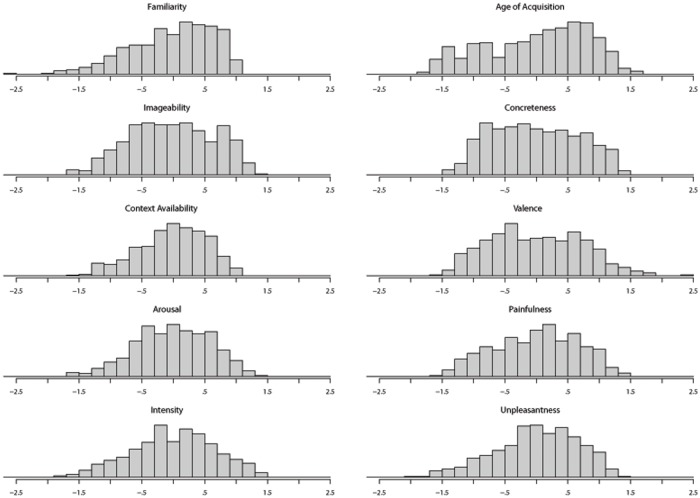
Variables distribution. Distribution of the variables in the final dataset.

We computed the reliability of the data for each variable by calculating the average split–half correlation over 1,000 random replicates, separately for each of the 20 questionnaires. Overall, the results showed a very strong reliability of the measures (Table 4 and Fig 3). The mean correlation value of each variable was very high, ranging from a minimum of r = .87 for Context Availability to a maximum of r = .98 for AoA. The mean correlation value of all the variables was M = .94 (SD = .03) suggesting that the collected ratings are highly reliable. Context Availability fared a little worse than the other variables, perhaps because it depends heavily on experience that is likely to vary quite substantially across participants. Because scores were standardized within participants, they are all reported on the same scale (z scores). Most variables had a rather symmetrical distribution, reasonably well centered on their mean and median (Fig 4). This was particularly true for Concreteness, Valence, Arousal, Pain-relatedness, and Intensity. Familiarity was quite left-skewed instead, not surprisingly given that the database includes several stimuli belonging to a medical jargon that may be rather unfamiliar to many participants. In addition, we cannot exclude that this result may also reflect the tendency to feel more familiar with pro-social and benevolent communication (*Linguistic positivity bias*, [7,94,124–126]). Overall, all the variables seemed quite well suited to investigate their effects on behavior with enough statistical power across their entire distribution.

**Table 4:** Correlation values for each variable resulting from the average split–half correlation for each questionnaire.

	Familiarity	Age of Acquisition	Imageability	Concreteness	Context Availability	Valence	Arousal	Pain-relatedness	Intensity	Unpleasantness
r	.91**	.98**	.94**	.95**	.87**	.97**	.92**	.95**	.95**	.95**

**p < .01

### Gender differences

We conducted t-tests to compare the scores of male and female participants. As shown in Table 5A, we did not find any significant differences suggesting that male and female participants rated pain words similarly. That ratings of male and female participants did not differ is also confirmed by the significantly high positive correlations of the ratings of female and male participants for all the variables [Familiarity (r = .884, p < .001), AoA (r = .963, p < .001), Imageability (r = .906, p < .001), Concreteness (r = .917, p < .001), Context Availability (r = .826, p < .001), Valence (r = .941, p < .001), Arousal (r = .846, p < .001), Pain-relatedness (r = .904, p < .001), Intensity (r = .899, p < .001), Unpleasantness (r = .918, p < .001)].

**Table 5:** Descriptive statistics and t-test concerning the ratings provided by male and female responders.

ALL WORDS									
	Males		Females		95% CI for Mean Difference				
	M	SD	M	SD	Inf.	Sup.	t	df	p
Familiarity	4.76	1.19	5.12	1.03	-0.02	0.03	0.26	510	.79
Age of Acquisition	4.68	1.40	4.66	1.40	-0.02	0.02	-0.06	510	.96
Imageability	4.86	1.29	4.86	1.27	-0.03	0.03	-0.01	511	.99
Concreteness	4.28	1.44	4.44	1.42	-0.02	0.03	0.04	511	.97
Context Availability	4.85	1.00	5.00	0.92	-0.03	0.03	0.02	511	.98
Valence	-1.09	0.98	-1.26	0.98	-0.02	0.03	0.22	509	.83
Arousal	4.14	1.14	4.32	1.11	-0.03	0.03	-0.01	511	.99
Pain-relatedness	4.15	1.46	4.41	1.40	-0.03	0.03	-0.04	511	.97
Intensity	51.19	21.46	55.20	21.28	-0.03	0.03	-0.01	511	.99
Unpleasantness	57.41	21.64	60.60	21.11	-0.02	0.02	-0.02	511	.98
PHYSICAL PAIN WORDS									
	Males		Females		95% CI for Mean Difference				
	M	SD	M	SD	Inf.	Sup.	t	df	p
Familiarity	4.78	1.20	5.13	1.04	-0.03	0.03	0.11	438	.91
Age of Acquisition	4.64	1.43	4.65	1.43	-0.02	0.02	0.37	438	.71
Imageability	4.91	1.30	4.90	1.31	-0.03	0.02	-0.24	439	.81
Concreteness	4.40	1.47	4.55	1.45	-0.03	0.02	-0.57	439	.57
Context Availability	4.85	1.01	4.99	0.93	-0.03	0.03	0.06	439	.95
Valence	-0.97	0.97	-1.14	0.97	-0.02	0.03	0.11	437	.91
Arousal	4.14	1.15	4.28	1.14	-0.05	0.01	-1.35	439	.18
Pain-relatedness	4.10	1.49	4.34	1.42	-0.04	0.01	-0.93	439	.35
Intensity	50.32	21.82	53.83	21.60	-0.05	0.01	-1.41	439	.16
Unpleasantness	55.38	21.63	58.55	21.19	-0.03	0.02	-0.38	439	.70
SOCIAL PAIN WORDS									
	Males		Females		95% CI for Mean Difference				
	M	SD	M	SD	Inf.	Sup.	t	df	p
Familiarity	4.66	1.13	5.12	0.94	-0.06	0.10	0.42	71	.68
Age of Acquisition	4.92	1.21	4.76	1.19	-0.08	0.03	-1.00	71	.32
Imageability	4.51	1.14	4.62	0.99	-0.05	0.09	0.58	71	.56
Concreteness	3.57	0.96	3.77	0.97	-0.01	0.12	1.57	71	.12
Context Availability	4.86	0.99	5.05	0.87	-0.07	0.06	-0.10	71	.92
Valence	-1.85	0.61	-2.00	0.63	-0.05	0.07	0.30	71	.77
Arousal	4.12	1.06	4.55	0.90	0.04	0.22	2.75	71	.01
Pain-relatedness	4.47	1.21	4.83	1.17	0.01	0.15	2.17	71	.03
Intensity	56.51	18.37	63.58	17.05	0.04	0.20	3.11	71	.00
Unpleasantness	69.80	17.18	73.16	15.68	-0.03	0.09	0.90	71	.37

It should be noted that also the original ANEW study [85] did not report any significant gender difference. In the Italian adaptation of the ANEW instead, Montefinese et al. [7] did find a significant gender difference on Arousal ratings, although the ratings were highly correlated (note that we did not test Dominance for which Montefinese et al. also reported a significant gender difference).

To further investigate potential gender differences, we also analyzed separately the ratings provided by female and male responders to physical and social pain words (Table 5B and 5C, respectively). Three significant differences emerged, all concerning social pain words. Female participants provided higher ratings of Arousal than male participants (see also [7]). In addition, female participants rated social pain words as more associated to pain and conveying more intense pain than male responders.

Table 5 refers to all the words together, as well as to physical pain words and social pain words alone.

### Hierarchical clustering analysis

**Fig 5:**
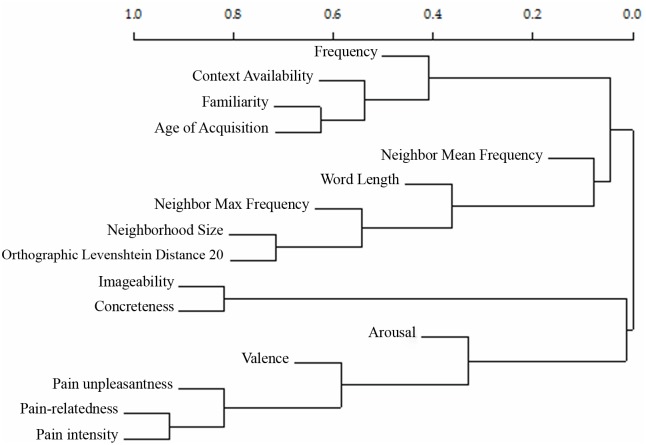
Hierarchical Clustering Analysis dendrogram. Dendrogram resulting from the Hierarchical Clustering Analysis of the 16 variables.

We also conducted a Hierarchical Clustering Analysis (HCA; Fig 5; [127]) that is ideal for exploring the correlational structure of the 16 measures used in this study. Hierarchical Clustering Analysis (HCA) is the general name of a family of techniques aimed at unveiling the underlying structure of a multivariate dataset by displaying it in a tree-like format [127]. HCA has the advantage of bringing out the main clusters in the data more clearly [128] and is particularly well suited to explore the correlational structure of a large number of measures. The dendrogram resulting from the HCA (Fig 5) shows that the highest split separates the lexical variables, the sub-lexical variables, Familiarity, AoA, and Context Availability on the one hand, from affective and pain-related variables, Imageability and Concreteness on the other hand. Within the former branch, Familiarity, AoA, and Context Availability cluster together, presumably because familiar words often are also acquired earlier and easier to contextualize. Word frequency (Zipf) stands on the top of this cluster. Another cluster is formed by distributional variables such as Neighbor Mean Frequency, Word Length, Neighbor Max Frequency, NSize, and OLD20. Interestingly, NSize and OLD20 are recognized as different metrics for the same construct (which they are indeed; e.g., [120]). It is not entirely clear what psychological construct this cluster may tap on. One possibility is that the core of the cluster is represented by Word Length, which strongly determines the features of a word’s lexical neighborhood. Within the second main branch, there is a cluster containing Imageability and Concreteness ratings, which is separated from the cluster relative to affective and pain-related variables. Interestingly, the structure of the affective and pain-related branch of the tree suggests that Pain-relatedness and Intensity are hardly separable. Differently, Unpleasantness stands alone, emerging as a distinct variable, albeit strongly correlated with the other two pain-related variables. That Intensity and Unpleasantness stand separately is consistent with experimental studies on pain showing that these two variables can be dissociated since they reflect two distinct components of pain (the sensory-discriminative component and the affective-motivational component, respectively) [129,130].

### Partial correlation analyses

**Fig 6:**
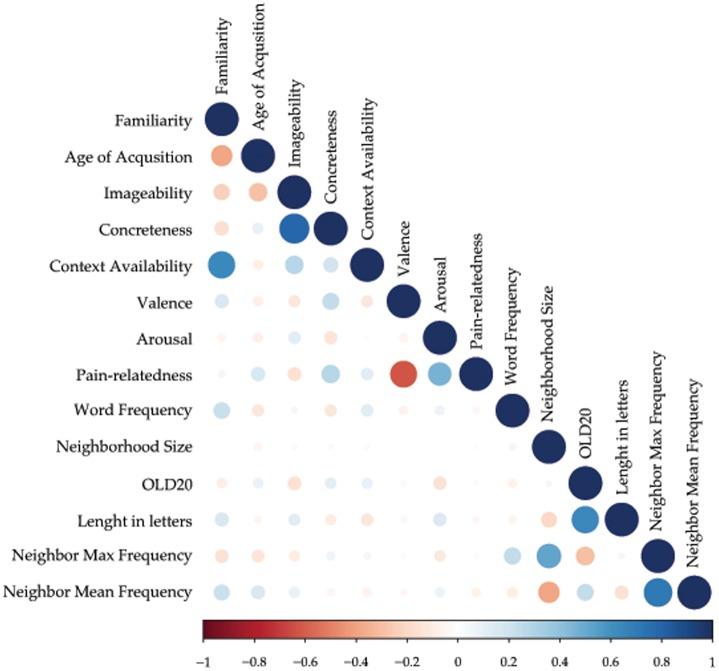
Partial correlations among all the variables. The dot color indicates the direction of the correlation (blue for direct, red for inverse) and the size and transparency its strength.

In what follows, we describe the results of the partial correlations among the variables (Fig 6 and Table 6). To avoid the problem of multicollinearity among Pain-relatedness, Intensity and Unpleasantness (r > .9), in these analyses we only used Pain-relatedness ratings. Moreover, given the high number of comparisons carried out (i.e., 91), we used a Bonferroni-corrected α value of .05/91 ≈ .0006. Finally, we present the results of separate one-way ANOVAs on the mean ratings of each variable for nouns, adjectives, and verbs and then for physical and social pain words.

**Table 6:** Partial correlations among all the variables.

	1	2	3	4	5	6	7	8	9	10	11	12	13	14
1. Familiarity	-	−.38*	−.22*	−.17*	+.64*	+.16	−.06	+.04	+.23*	−.00	−.09	+.16*	−.15	+.21*
2. Age of Acquisition		-	−.29*	+.10	−.08	−.08	−.08	+.16*	−.12	−.05	+.09	−.05	−.13	+.16
3. Imageability			-	+.78*	+.28*	−.11	+.12	−.15	+.03	−.02	−.15	+.11	−.10	+.10
4. Concreteness				-	+.19*	+.24*	−.14	+.28*	−.12	+.03	+.10	−.10	+.06	−.05
5. Context Availability					-	−.11	−.02	+.12	+.12	−.03	+.10	−.12	+.04	−.06
6. Valence						-	−.06	−.62*	−.06	+.00	+.03	−.03	+.03	−.04
7. Arousal							-	+.46*	+.07	+.00	−.14	+.14	−.10	+.08
8. Pain-relatedness								-	−.04	+.01	+.02	−.03	+.02	−.06
9. Zipf									-	+.04	−.06	−.06	+.24*	−.09
10. N										-	+.03	−.20*	+.52*	−.39*
11. OLD20											-	+.64*	−.29*	+.23*
12. Letters												-	+.03	−.16
13. MaxFreqN													-	+.71*
14. MeanFreqN														-

Abbreviations refer to the following variables: Subtlex-IT Frequency (Zipf), Neighborhood Size (N), Orthographic Levenshtein Distance 20 (OLD20), Neighbor max frequency (MaxFreqN), Neighbor mean frequency (MeanFreqN).

*p < .0006.

#### Partial correlations among psycholinguistic variables

Partial correlation analyses (Table 6) revealed that more familiar words are learnt earlier in life (r = -.38) and are more prone to elicit a context (r = .64). In fact, Familiarity inversely correlates with AoA and positively correlates with Context Availability [31,131–133]. The more familiar pain words are, the less imaginable and concrete are (r = -.22 and r = -.17, respectively). Admittedly, we do not have an explanation for the significant inverse correlations between Familiarity and Imageability, and between Familiarity and Concreteness, which are inconsistent with what is typically reported in the literature on affective words (e.g., [31,67,132,133]; but see [131]) (we return on this point in the Conclusions). Further analyses conducted on the three word classes and on physical and social pain separately are shown in S1 Table and revealed that these two inverse correlations are statistically significant only for nouns (and not for adjectives and verbs) and specifically only for physical pain nouns. One possibility is that these inverse correlations reflect the specific type of affective nouns tested in this study. In fact, the words that we most often use to convey physical pain include a variety of nouns as, for instance, names of syndrome and illness (e.g., *gastrite*, *gastritis*) and generic terms (e.g., *acciacco*, *infirmity*) that are hardly concrete and imageable.

Frequency is significantly correlated only with Familiarity [31,131,133] in that the more frequent a word is, the more familiar it is rated (r = .23), quite unsurprisingly. Words learnt earlier in life are also rated as more imaginable (r = -.29), in line with the literature [31,108,131,133]. Again in line with the literature [113,131,134,135], the more a pain word is concrete, the more it is imageable and prone to elicit a context (r = .28 and r = .19, respectively). Positive correlations between Imageability and Concreteness for affective words have been reported in a variety of languages, including English [104,135], Chinese [136], European Portuguese [89], French [137], and Spanish [138]. Finally, longer words are rated as more familiar and with smaller neighborhoods and higher OLD20 values.

#### Partial correlations between affective and pain-related variables

**Fig 7:**
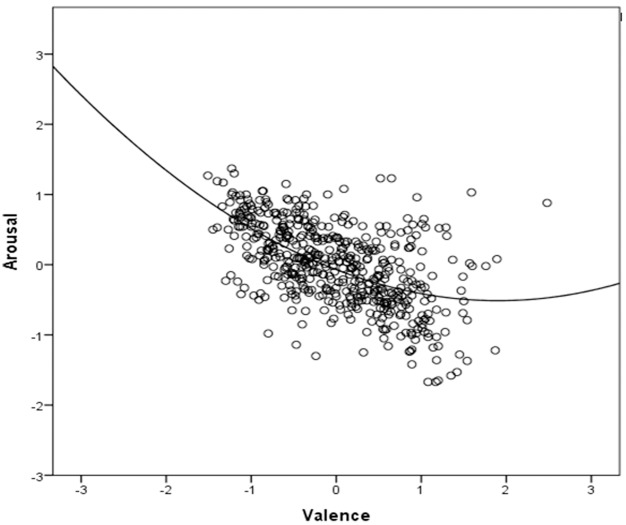
Partial scatterplot. Partial scatterplot of mean values in the Valence and Arousal dimensions, along with the quadratic regression line (R^2^ = .33).

According to the literature on affective words [7,32,70,71,99,139,140], valence and arousal ratings typically exhibit a U-shaped relationship whereby highly valenced words (both positive and negative) also have higher arousal ratings than neutral words. The bivariate correlation between Valence and Arousal ratings of pain-related words reveals a significant linear rather than a quadratic relationship (r = -.56). The bivariate correlation between Valence and Arousal ratings of pain-related words (Fig 7) reveals a significant linear rather than a quadratic relationship (r = -.56), possibly representing the negative portion of the classic U-shaped relationship.

This database is about pain words which of course moves the valence distribution towards its negative end. However, this correlation is not significant anymore after controlling for the effects of psycholinguistic and pain-related variables. Partial correlations, instead, reveal that the more a word is associated to pain, the more negative and arousing it is. In fact, Pain-relatedness inversely correlates with Valence and positively correlates with Arousal. This is consistent with studies on emotionally-laden words showing that an increase in negative valence is often associated to an increase in arousal (e.g., [7,8,69,71,88]).

#### Partial correlations among psycholinguistic, affective, and pain-related variables

The more positive a word is, the more concrete it is rated, as shown by a positive correlation between Valence and Concreteness. This result is consistent with prior studies showing a joint effect of valence and concreteness on word recognition in a variety of tasks (for an overview, see [141]). Finally, the more a word is associated to pain, the more it is rated as concrete and acquired later in life, as shown by positive correlations between Pain-relatedness and AoA, and Pain-relatedness and Concreteness.

### Differences among word classes

Our database is composed by 42.6% of adjectives, 38.9% of nouns, 14.6% of verbs and 3.9% of ambiguous words (i.e., adjectives that can also be used as nouns). Since grammatical class is known to affect linguistic processing, and specifically that of affective words [98], we conducted separate by-item one-way ANOVAs on each variable with Word Class (Adjectives vs. Nouns vs. Verbs) as a between-item factor.

The one-way ANOVA on AoA reveals a statistically significant difference among word classes [F (2,488) = 9.564, p < .001, η^2^ = .038]. Post-hoc comparisons (with the Tukey HSD test) show that verbs (M = 4.13, SD = 1.46) are learnt significantly earlier than both nouns (M = 4.65, SD = 1.38, p = .015) and adjectives (M = 4.92, SD = 1.3, p < .001). This is likely to reflect the specific semantic domain tested in this study. In fact, while many of the nouns referring to pain concern events or experiences predominantly occurring in adulthood (e.g., *tremore*, *tremor*; *abbandono*, *neglect*), verbs describe actions that are rather common in the childhood (e.g., *scivolare*, *to slip*; *cadere*, *to fall*; *graffiare*, *to scratch*).

One-way ANOVAs show significant word class effects also on Imageability [F(2,489) = 106.105, p < .001, η^2^ = .303], Concreteness [F (2,489) = 138.229, p < .001, η^2^ = .361], and Context Availability [F(2,489) = 54.733, p < .001, η^2^ = .183]. In fact, adjectives are rated as significantly less imaginable, concrete and also less prone to elicit a context than nouns and verbs.

One-way ANOVAs on Valence [F(2,487) = 39.592, p < .001, η^2^ = .14] and Arousal [F(2,489) = 29.274, p < .001, η^2^ = .11] show significant word class effects as well. Adjectives are rated as more positive and less arousing than nouns and verbs. This may reflect the fact that a consistent number of our adjectives can be used to modify pain-unrelated nouns as well (e.g., *grande*, *big*, *acuto*, *acute*). In fact, 78 of the 218 adjectives are rated as weakly or not at all associated to pain (Pain-relatedness < 3). Moreover, verbs are rated as significantly more arousing than nouns (p < .001), reflecting the action-oriented nature of most of our verbs.

One-way ANOVAs on Pain-relatedness [F(2,489 = 57.79, p < .001, η^2^ = .191], Intensity [F (2,489) = 44.354, p < .001, η^2^ = .154] and Unpleasantness [F(2,489) = 36.806, p < .001, η^2^ = .131] again reveal significant effects of word class. Unsurprisingly, adjectives are judged as significantly less pain-related and conveying a less intense and unpleasant pain than nouns and verbs. Again, this may reflect the fact that many of our adjectives have a general semantic scope (e.g., *grande*, *big*; *immenso*, *immense*). ANOVA on Familiarity does not reveal any significant differences among the three word classes [F(2,489) = 1.114, p = .329, η^2^ = .005].

Partial correlations for nouns, adjectives, and verbs are reported in S1 Table.

### Differences between words conveying physical and social pain

In order to understand whether the psycholinguistic and affective properties of physical and social pain words differ, we conducted by-item one-way ANOVAs on each variable with Type of Pain (Physical vs. Social) as a between-item factor.

One-way ANOVAs on Concreteness [F(1,510) = 21.112, p < .001, η^2^ = .04], Valence [F(1,508) = 52.77, p < .001, η^2^ = .094], Pain-relatedness [F(1,510) = 6.352, p = .012, η^2^ = .012], Intensity [F(1,510) = 10.136, p = .002, η^2^ = .019], and Unpleasantness [F(1,510) = 28.377, p < .001, η^2^ = .053] yield statistically significant differences. Specifically, the words conveying social pain are rated as less concrete, but more negative, than the words conveying physical pain. Interestingly, participants rate social pain words as more associated to pain, and conveying a more intense and unpleasant pain, than physical pain words.

ANOVAs on Familiarity [F(1,510) = .001, p = .97, η^2^ = .000], AoA [F(1,510) = 2.720, p = .397, η^2^ = .001], Imageability [F(1,510) = 3.498, p = .062, η^2^ = .007], Context Availability [F(1,510) = .436, p = .509, η^2^ = .001], and Arousal [F(1,510) = 2.104, p = .148, η^2^ = .004] do not reveal any significant differences between physical and social pain.

## Conclusions

The aim of the present study was twofold. First, we assessed the psycholinguistic, affective, and pain-related characteristics of Italian words conveying physical and social pain providing a normed lexicon of pain. Second, we explored the relationships among these variables unveiling important aspects of the lexico-semantic architecture underlying the Italian pain lexicon. To these aims, we collected ratings for psycholinguistic, affective and pain-related variables, as well as distributional data, for 512 words expressing physical and social pain. These norms respond to the need for normed stimuli to be used in the experimental research on pain and on negative affect in Italian.

We carried out a Hierarchical Clustering Analysis (HCA) to explore the structure underlying the correlations among the 16 variables measured in this study. Two interesting results emerge from the HCA. The first is that pain-related variables cluster separately from all the other variables. The second interesting result concerns the organization of pain-related variables that shows two different clusters: Unpleasantness, that clusters by itself pointing to the affective-motivational dimension of pain, and Intensity and Pain-relatedness that cluster together pointing to the sensory-discriminative dimension of pain.

In line with prior studies on the affective lexicon, we found that the pain words acquired earlier in life are also more familiar and imageable [31,67,131,133,142], and that more familiar words are also more easily associated to specific contexts. More imaginable words are also rated as more concrete [113,131,134,135] and more prone to elicit a context. At variance with the literature [108,131], we found that the more physical pain nouns are familiar, the less imaginable and concrete they are rated. Admittedly, we do not yet have an explanation for these results. One possibility is that they may reflect the semantic heterogeneity of the nouns of this corpus that include medical terms (e.g., *gastrite*, *gastritis*), illness generic nouns and lay person pain words (e.g., *acciacco*, *infirmity*) not easily classifiable as imageable and/or concrete. In addition, responders may know the names of painful events, states or illnesses they have never directly experienced hence diminishing their ability to decide how much they are concrete and to image them. Even the words *pain* or *disease* refer to generic, intangible, and poorly delineated experiences, not directly observable [11], that are likely to be considered scarcely concrete and/or imageable.

Verbs conveying actions that may cause pain, or represent antecedents of pain experiences, are judged to have been acquired earlier than adjectives and nouns. This suggests that the development of a more sophisticated pain-related lexicon emerges as we grow up. This lexicon is used to convey a broad range of painful experiences, including those producing social pain. This is confirmed by the positive correlation between Pain-relatedness and AoA that reveals that the words more associated to pain are also judged to be learnt later in life.

Social pain words are rated as more negative and pain-related than physical pain words, and as reflecting more intense and unpleasant pain experiences than physical pain words. This is likely to reflect the relatively young age of our responders for whom social pain could represent a more salient and frequent experience than physical pain. In fact, 17.8% of the responders answered that they currently suffer of chronic pain and 5.1% of chronic pain in the past. These percentages are important but in any case lower than the mean incidence of chronic pain in the Italian population that concerns the 26% of Italians [143]. However, since the question was phased rather generically without specifically listing what could count as “chronic pain”, or the types of experienced chronic pain, we cannot be sure that indeed it was selected by responders suffering chronic pain as defined in the clinical literature. In any case, since a qualitative inspection of the results of the two subsets of participant (i.e., responders with and without actual/past chronic pain) did not suggest any differences in the distribution of the ratings of the variable tested, they were analyzed all together. However, a possible important effect of age on physical vs. social pain perception may not represent the whole story. In fact, a wealth of studies about the subjective impact of social pain has documented that often this is considered as much threatening and important as physical pain. Notably, nearly three out of four people listed the loss of a close relationship for death or relationship break-up as the “single most negative emotional event” of their lives [56,144]. A study administering the same questionnaires to older participants (41–70 years) is currently in progress to clarify whether the higher negativity and Pain-relatedness of social pain words indeed depend on the age of responders.

One might wonder whether suffering or having suffered of chronic pain may have a general effect on the ratings provided for physical pain words. Assessing whether participants in the study have, or have had, painful experiences, either physical or social, would be crucial to clarify this point. However, as we mentioned, we only asked generically if the responders suffered or had ever suffered of chronic pain and we did not investigate at all whether responders suffered of had ever suffered of social pain. Admittedly, this is an important limitation of this study. In fact, the possibility exists that both forms of pain may affect the ways in which we linguistically categorize and evaluate pain. We are currently running a study on cancer patients where we administer them an adapted form of the WOP. This could clarify whether a condition of severe oncological pain affects the semantic of pain. We expect that this may be the case since pain is intimately associated with alterations of physiological and psychological processes of pain perceptions and pain-related behaviors [145,146].

The biological gender of participants does not seem to affect the results of our study, differently from what was found for Italian affectively-laden words by Montefinese et al. [7], although only for arousal. However, as Montefinese et al. clarified, these gender differences are moderated by the high correlation between male and female ratings of arousal found in the study. A growing body of research about the role of gender differences in medical language and communication has reported gender differences in the affective and social content of symptoms descriptions, willingness to report pain, and words used to describe pain [147,148]. These differences have been linked to psycho-social gender roles. However, these gender differences may not necessarily lead to different ratings of the psycholinguistic and affective variables tested in this study [149]. In addition, we cannot exclude the possibility that gender differences in pain communication could emerge once pain and illness have been consistently experienced, usually later in life. However, due to the online recruitment of responders that reflected the preponderance of female students, we did not have the same number of male and female participants. Although we cannot exclude that this may have influenced the lack of significant gender differences, it should be noted that other more gender-balanced studies on the affective lexicon did not find gender differences either.

Pain words belong to the realm of negative words. Interestingly, our results suggest that not all pain words seem to be negative alike. For instance, the words associated to labor pain (e.g., *partorire*, *to give birth*; *doglia*, *labor pain*) are rated as extremely intense and unpleasant but with a predominantly positive Valence. Interestingly, these word ratings are similar to the ratings of Intensity, Unpleasantness and Valence reported in the literature on labor pain. In fact, when asked to evaluate their childbirth experience, women rated it as extremely high in Intensity, but lower in Unpleasantness than other types of pain, and having a positive Valence [150].

Consensus exists that stimuli are automatically evaluated in terms of their affective valence [151,152] along a negative-to-positive valence gradient [22,116,153,154]. So far, studies on valenced words have predominantly treated negative words as a unitary category. However, recently it has been suggested that negative words may not represent a unitary category but rather they may differ based on their specific semantic content [155,156]. For instance, a recent brain-imaging meta-analysis has shown that the brain did not treat negative stimuli (be they words or images) as a unified class [157–159]. One can speculate that pain words may represent a domain with a specific status among negatively valenced words due to the high relevance of pain experiences in everyday life and for survival. Future studies devoted to test this aspect are required before one can draw any firm conclusions.

WOP provides norms about the specific part of our lexicon in that convey physical and social pain. We obviously see this as an important strength of this work. However, this also determined the presence of a few positively valenced words in our database. This limitation is mitigated by the fact that 78 of the 218 adjectives of WOP can be used to modify pain-related as well as pain-unrelated nouns (e.g., *immenso*, immense, *grande*, big, *infinito*, infinite). In fact, 51 out of these 78 adjectives were rated as positive together with the noun *parto* (*delivery*) and the verb *partorire* (*to deliver*). Nine adjectives were rated as neutral, together with the verbs *grattare* (*to scratch*) and *stringere* (*to tighten*). The general Valence distribution of our stimuli is indeed a little skewed towards the negative end (mean = -.9, median = -1.3), but covers the entire range of possible values (min = -2.97, max = +2.52). A similar consideration applies to Pain-relatedness that may be expected to peak very narrowly around high values; but it did not. In fact, Pain-relatedness ranged from 1.16 to 6.83, with a mean value of 4.34 and median value of 4.43, mostly thanks to adjectives. Therefore, although the database is obviously tight to the specific investigation of pain words, it does provide a wider spectrum of stimuli.

Finally, we acknowledge that we had a different number of observations per cell for some stimuli and that this may represent a problem. However, our ratings were provided by at least 31 responders which represents a reasonable number of observations compared to other databases (for instance, the Italian version of ANEW provides affective ratings from at least 31 participants and psycholinguistics ratings from 20 participants).

To the best of our knowledge, this is the first descriptive study on the psycholinguistic, affective, and pain-related characteristics of physical and social pain words. This normative study provides a useful tool that may enable researchers to use highly controlled stimuli in experimental studies on physical and social pain as well as on language and negative affect.

## Supporting information

S1 Text Original, Italian survey instruction are provided together with the English translation. (DOCX)

S1 Fig Example of the Rodriguez and Laio clustering procedure. An example of the Rodriguez and Laio clustering procedure using Familiarity ratings (on a 7-point scale) for the words “correlation” and “variance” given by ten participants (from subject 1 to subject 10). They are represented as points in a two–dimensional space, and their position is defined by their ratings. Subjects 1 to 4 (s1–s4, in green color) gave consistent, high judgments; subjects 5 to 8 (s5–s8, in blue color) also gave consistent, low judgments. Conversely, subjects 9 and 10 (s9–s10, in red color) provided highly idiosyncratic responses, as indicated by their isolated position on the graph.
(DOCX)

S1 Table Partial correlations among all the variables of interest considering the three word classes and physical and social pain separately. Table 1A refers to partial correlations for nouns. Table 1B refers to partial correlations for adjectives. Table 1C refers to partial correlations for verbs. Table 1D refers to partial correlations for physical pain words. Table 1E refers to partial correlations for social pain word. Table 1F refers to partial correlations for physical pain nouns. Abbreviations: Subtlex-IT Frequency (Zipf), Neighborhood Size (N), Orthographic Levenshtein Distance 20 (OLD20), Neighbor Max Frequency (MaxFreqN), Neighbor Mean Frequency (MeanFreqN).
(DOCX)
