# Supplementary material for: The psycholinguistic and affective structure of words conveying pain
Source: PLoS One. 2018 Jun 29;13(6):e0199658. doi: 10.1371/journal.pone.0199658 (PMC6025857; doi:10.1371/journal.pone.0199658)
Supplement: S1 Text — (DOCX) [file pone.0199658.s001.docx]

**Survey instruction in Italian**

Buongiorno. Questo questionario si propone di raccogliere informazioni sul modo in cui le persone parlano del dolore. La sua compilazione richiede indicativamente 45 minuti. Di seguito troverai un elenco di parole che riguardano il dolore:

- aggettivi utilizzati per descrivere il dolore (es. pungente, persistente,...);

- sostantivi che indicano stimoli/eventi che possono arrecare dolore (es. ago, malattia, lutto,...) o aspetti del dolore (es. patimento, sofferenza,...);

- verbi relativi ad azioni che possono arrecare dolore (es. bruciare, ferire,...).

Per ciascuna parola dovrai esprimere una valutazione seguendo le istruzioni che troverai di volta in volta. Se non hai mai sentito una parola oppure se l’hai già sentita ma non ne conosci il significato, barra la casella NON CONOSCO. Ti chiediamo di rispondere a tutte le domande. Non avere fretta nel compilarlo, ma cerca di essere accurato e spontaneo.

**MADRELINGUA** (Italiana, Non italiana)

**SESSO** (Maschio, Femmina)

**ETÀ**

**TITOLO DI STUDIO**

**SOFFRI DI UNA QUALCHE FORMA DI DOLORE CRONICO O DI CEFALEE INTENSE E RIPETUTE?** (Sì, No, Ne ho sofferto in passato)

**FAMILIARITÀ -** Tra le parole che utilizziamo, ascoltiamo e leggiamo, ne esistono alcune che incontriamo (utilizziamo, ascoltiamo o leggiamo) molto spesso (estremamente familiari) e altre che non incontriamo quasi mai (per niente familiari). Per esempio, la parola BICCHIERE è molto familiare, mentre la parola BALESTRA è poco familiare perché raramente la si incontra. Valuta per ciascuna delle seguenti parole quanto ti è familiare in una scala che va da 1 (per niente familiare) a 7 (estremamente familiare).

**ETÀ DI ACQUISIZIONE -** Valuta l'età a cui pensi di aver appreso una parola. Per esempio, la parola LAVAGNA potresti averla appresa intorno ai 5 anni, mentre la parola PAPPA entro i primi due anni.

**IMMAGINABILITÀ** - Le parole differiscono quanto alla loro capacità di evocare una immagine mentale corrispondente. Alcune parole generano facilmente e rapidamente un'immagine mentale (estremamente immaginabili) mentre altre riescono a farlo solo con difficoltà (per niente immaginabili). Per esempio, la parola MANDARINO evoca facilmente un'immagine, mentre la parola CONCETTO difficilmente riesce a evocare qualcosa. Valuta per ciascuna delle seguenti parole il grado di immaginabilità in una scala che va da 1 (per niente immaginabile) a 7 (estremamente immaginabile).

**CONCRETEZZA** - Alcune parole si riferiscono a persone, luoghi, cose, azioni e situazioni che possono essere facilmente percepite attraverso i sensi (cioè sono estremamente concrete), mentre altre parole non si riferiscono a qualcosa percepibile direttamente attraverso i sensi (cioè non sono per niente concrete). Per esempio, la parola LIBRO è estremamente concreta, in quanto si riferisce a un oggetto che vediamo e tocchiamo e di cui abbiamo esperienza attraverso i sensi, mentre la parola TEOREMA non è per niente concreta perché non è possibile averne esperienza attraverso i sensi. Valuta per ciascuna delle seguenti parole il grado di concretezza in una scala che va da 1 (per niente concreta) a 7 (estremamente concreta).

**DISPONIBILITÀ DI CONTESTO** - In alcuni casi è molto semplice pensare a un contesto o circostanza in cui potremmo sentire o usare una certa parola, mentre in altri casi può essere più difficile se non impossibile pensare a un contesto in cui

incontrare quella parola. Per esempio, mentre è facile pensare a un contesto per la parola PIANTA, non è altrettanto facile pensare a un contesto per la parola PRETESTO.

Valuta per ciascuna delle seguenti parole il grado di disponibilità di un contesto in una scala che va da 1 (contesto per niente disponibile) a 7 (contesto estremamente disponibile).

**ASSOCIAZIONE AL DOLORE** - Valuta per ciascuna delle seguenti parole quanto la associ al dolore inteso in senso ampio su una scala che va da 1 (per niente associata) a 7 (estremamente associata).

**INTENSITÀ** - Le parole che seguono possono descrivere diversi aspetti associati all'esperienza del dolore (sia fisico che psicologico). Per ognuna devi valutare l'intensità del dolore che essa trasmette. Sposta il cursore posizionandolo nel punto che corrisponde a questa tua stima di intensità considerando che la scala va da "Per niente intensa" (la parola non trasmette alcuna intensità di dolore) a "Estremamente intensa" (la parola trasmette la massima intensità di dolore possibile).

**SPIACEVOLEZZA** - Le parole che seguono possono descrivere diversi aspetti associati all'esperienza del dolore (sia fisico che psicologico). Per ognuna devi valutare quanto spiacevole è il dolore che la parola descrive. Sposta il cursore posizionandolo nel punto che corrisponde a questa tua stima considerando che la scala va da "Per niente spiacevole" a "Estremamente spiacevole".

**VALENZA** - La valenza descrive quanto il significato veicolato da una parola è negativo o positivo. Per esempio, per la maggior parte delle persone la parola GUERRA è negativa, mentre la parola ABBRACCIO è positiva. Altre parole, come SEDIA, non sono né negative né positive. Valuta quanto negativo o positivo è per te il significato veicolato dalle seguenti parole in una scala che va da -3 (estremamente negativo) a +3 (estremamente positivo).

**ATTIVAZIONE** - L'attivazione descrive quanto il significato veicolato da una parola si riferisce a qualcosa di molto attivante ed eccitante o, al contrario, a qualcosa che non attiva, che smorza o calma. Per esempio, per la maggior parte delle persone la parola ABETE evoca un senso di calma e di inattivazione, mentre la parola URAGANO evoca un senso di attivazione ed energia. Altre parole, come FILO, non trasmettono né un senso di attivazione, né un senso di inattività. Valuta quanto le seguenti parole trasmettono un significato legato all'attivazione e all'energia oppure all'assenza di attivazione ed energia in una scala che va da 1 (per niente attivante) a 7 (estremamente attivante).

**Survey instruction in English**

Good morning. This survey aims to collect information about how people talk about pain. Its compiling takes about 45 minutes. You will be presented with a list of words related to pain:

- adjectives used to describe pain (e.g., stinging, continuous);

- names indicating stimuli/events that may cause pain (e.g., needle, illness, grief) or pain aspects (e.g., tribulation, suffering);

- verbs related to painful actions (e.g., to burn, to wound).

Please, rate each word following the instructions that you will find each time. If you have never heard a word, or if you have heard it but you don’t know its meaning, check the UNKNOWN option. Please, answer all the questions. Don’t be hurried and try to be accurate and spontaneous.

**MOTHERTONGUE**

**GENDER** (Male, Female)

**AGE**

**EDUCATION LEVEL**

**DO YOU SOFFER OF CHRONIC PAIN OR OF INTENSE AND REPEATED HEADACHES?** (Yes, No, In the past)

**FAMILIARITY** - Among all words we use, listen and read, there are some that we use, listen, and read more frequently (extremely familiar) and others that we use, listen, and read less frequently (not familiar at all). For example, the word GLASS is extremely familiar, whereas the word CROSSBOW is not familiar at all. Rate the Familiarity of each of the following words on a scale ranging from 1 (not at all familiar) to 7 (extremely familiar).

**AGE OF ACQUISITION** - Evaluate the age at which you think you have learnt the following words. For example, you could have learnt the word BLACKBOARD at about 5yrs, whereas the word DIN-DINS by 2yrs.

**IMAGEABILITY** - Words differ for the easiness they evoke a mental image. Some words evoke easily and quickly a mental image (extremely imaginable), whereas other words evoke it barely (not imaginable at all). For example, the word TANGERIN easily evokes a mental image, whereas the words CONCEPT barely does it. Rate the following words on the bases of their Imageability on a rating scale ranging from 1 (not at all imaginable) to 7 (extremely imaginable).

**CONCRETENESS** - Some words refer to people, places, actions and situations that may be easily perceived through our senses (i.e., they are extremely concrete), whereas other words do not refer to something perceivable through our senses (not concrete at all). For example, the word BOOK is extremely concrete, because it refers to an object that we can see and touch and that we can experience through our senses, whereas the word THEOREM is not concrete at all because we cannot experience it through our senses. Rate the following words on the bases of their Concreteness on a rating scale ranging from 1 (not at all concrete) to 7 (extremely concrete).

**CONTEXT AVAILABILITY** - Sometimes it is very easy to find out a context or a circumstance in which we could hear or use a word, whereas sometimes it could be very hard if not impossible. For example, it is easy to find out a context for the word PLANT, but it is not for the word PRETEXT.

Rate the following words on the bases of their context availability on a rating scale ranging from 1 (context not at all available) to 7 (context extremely available).

**PAIN RELATEDNESS** - Rate for the following words the degree to which you relate them to the pain, in its broader meaning, on a rating scale ranging from 1 (not related at all) to 7 (extremely related).

**INTENSITY** - The following words may describe aspects of the pain experience (both physical and psychological). Rate the intensity of the pain they convey by moving the slider arranging it in the corresponding position of the line, considering that it range from “Not intense at all” to “extremely intense”.

**UNPLEASANTNESS** - The following words may describe aspects of the pain experience (both physical and psychological). Rate the unpleasantness of the pain they convey by moving the slider arranging it in the corresponding position of the line, considering that it range from “Not at all unpleasant” to “extremely unpleasant”.

**VALENCE** - Valence describes the degree to which the meaning of a word is negative or positive. For example, for most of the people, the word WAR is negative, whereas the word HUG is positive. Other words, like CHAIR, are not negative, nor positive. Rate how much the meaning conveyed by the following words is negative or positive on a rating scale ranging from -3 (extremely negative) to +3 (extremely positive).

**AROUSAL** - Arousal describes the degree to which the meaning of a word refers to something arousing and exciting or to something not arousing, that calm down. For example, for most of the people, the word FIR evokes a sense of deactivation and calm, whereas the word HURRICANE evokes a sense of activation and energy. Other words, like STRING, dies not evoke a sense of activation nor of deactivation. Rate how much the meaning conveyed by the following words are arousing or not on a rating scale ranging from 1 (not at all arousing) to 7 (extremely arousing).
