# Supplementary material for: The psycholinguistic and affective structure of words conveying pain
Source: PLoS One. 2018 Jun 29;13(6):e0199658. doi: 10.1371/journal.pone.0199658 (PMC6025857; doi:10.1371/journal.pone.0199658)
Supplement: S1 Fig — An example of the Rodriguez and Laio clustering procedure using Familiarity ratings (on a 7-point scale) for the words “correlation” and “variance” given by ten participants (from subject 1 to subject 10). They are represented as points in a two–dimensional space, and their position is defined by their ratings. Subjects 1 to 4 (s1–s4, in green color) gave consistent, high judgments; subjects 5 to 8 (s5–s8, in blue color) also gave consistent, low judgments. Conversely, subjects 9 and 10 (s9–s10, in red color) provided highly idiosyncratic responses, as indicated by their isolated position on the graph. (DOCX) [file pone.0199658.s002.docx]

**S2_Figure1. Example of the Rodriguez and Laio clustering procedure.** An example of the Rodriguez and Laio clustering procedure using Familiarity ratings (on a 7-point scale) for the words “correlation” and “variance” given by ten participants (from subject 1 to subject 10). They are represented as points in a two–dimensional space, and their position is defined by their ratings. Subjects 1 to 4 (s1-s4, in green color) gave consistent, high judgments; subjects 5 to 8 (s5-s8, in blue color) also gave consistent, low judgments. Conversely, subjects 9 and 10 (s9-s10, in red color) provided highly idiosyncratic responses, as indicated by their isolated position on the graph.
