# Supplementary material for: The psycholinguistic and affective structure of words conveying pain
Source: PLoS One. 2018 Jun 29;13(6):e0199658. doi: 10.1371/journal.pone.0199658 (PMC6025857; doi:10.1371/journal.pone.0199658)
Supplement: S1 Table — Table 1A refers to partial correlations for nouns. Table 1B refers to partial correlations for adjectives. Table 1C refers to partial correlations for verbs. Table 1D refers to partial correlations for physical pain words. Table 1E refers to partial correlations for social pain word. Table 1F refers to partial correlations for physical pain nouns. Abbreviations: Subtlex-IT Frequency (Zipf), Neighborhood Size (N), Orthographic Levenshtein Distance 20 (OLD20), Neighbor Max Frequency (MaxFreqN), Neighbor Mean Frequency (MeanFreqN). (DOCX) [file pone.0199658.s003.docx]

**S3_Table1. Partial correlations among all the variables of interest considering the three word classes and physical and social pain separately.** Table 1A refers to partial correlations for nouns. Table 1B refers to partial correlations for adjectives. Table 1C refers to partial correlations for verbs. Table 1D refers to partial correlations for physical pain words. Table 1E refers to partial correlations for social pain word. Table 1F refers to partial correlations for physical pain nouns. Abbreviations: Subtlex-IT Frequency (Zipf), Neighborhood Size (N), Orthographic Levenshtein Distance 20 (OLD20), Neighbor Max Frequency (MaxFreqN), Neighbor Mean Frequency (MeanFreqN).

| Table 1A | Nouns | | | | | | | | | | | | | |
| --- | --- | --- | --- | --- | --- | --- | --- | --- | --- | --- | --- | --- | --- | --- |
|  | 1 | 2 | 3 | 4 | 5 | 6 | 7 | 8 | 9 | 10 | 11 | 12 | 13 | 14 |
| 1. Familiarity | - | -.31* | -.3* | -.07 | +.71* | -.02 | -.09 | -.04 | +.17 | -.03 | -.04 | +.1 | -.09 | +.15 |
| 2. Age of Acquisition |  | - | -.32* | +.12 | -.08 | -.12 | -.02 | +.09 | -.22 | -.09 | -.05 | .11 | -.07 | +.07 |
| 3. Imageability |  |  | - | +.78* | +.28* | -.19 | +.01 | -.18 | +.07 | -.03 | -.21 | +.29* | -.03 | +.04 |
| 4. Concreteness |  |  |  | - | +.09 | +.41* | -.06 | +.35* | -.2 | +.02 | +.15 | -.26 | +.01 | +.01 |
| 5. Context Availability |  |  |  |  | - | +.01 | +.03 | +.14 | +.09 | +.01 | +.07 | -.06 | +.03 | -.09 |
| 6. Valence |  |  |  |  |  | - | -.04 | -.68* | -.06 | -.00 | -.00 | +.02 | +.00 | -.00 |
| 7. Arousal |  |  |  |  |  |  | - | +.39* | +.1 | -.02 | -.06 | +.02 | -.04 | -.02 |
| 8. Pain-relatedness |  |  |  |  |  |  |  | - | +.06 | +.04 | +.09 | -.02 | +.08 | +.02 |
| 9. Zipf |  |  |  |  |  |  |  |  | - | +.01 | -.31* | +.02 | +.04 | -.06 |
| 10. N |  |  |  |  |  |  |  |  |  | - | +.00 | -.22 | +.45* | -.35* |
| 11. OLD20 |  |  |  |  |  |  |  |  |  |  | - | +.65* | -.45* | +.22 |
| 12. Letters |  |  |  |  |  |  |  |  |  |  |  | - | +.18 | -.17 |
| 13. MaxFreqN |  |  |  |  |  |  |  |  |  |  |  |  | - | +.72* |
| 14. MeanFreqN |  |  |  |  |  |  |  |  |  |  |  |  |  | - |

*p < .0006

| Table 1B | Adjectives | | | | | | | | | | | | | |
| --- | --- | --- | --- | --- | --- | --- | --- | --- | --- | --- | --- | --- | --- | --- |
|  | 1 | 2 | 3 | 4 | 5 | 6 | 7 | 8 | 9 | 10 | 11 | 12 | 13 | 14 |
| 1. Familiarity | - | -.33* | -.17 | -.2 | +.64* | +.16 | -.09 | +.06 | +.26* | +.02 | -.17 | +.25 | -.11 | +.18 |
| 2. Age of Acquisition |  | - | -.23 | +.06 | -.13 | +.02 | -.13 | +.24 | -.2 | -.03 | +.02 | -.05 | -.2 | +.22 |
| 3. Imageability |  |  | - | +.78* | +.28* | -.12 | +.17 | -.19 | -.19 | -.04 | +.02 | -.11 | -.09 | +.04 |
| 4. Concreteness |  |  |  | - | +.12 | +.24 | -.17 | +.29* | -.08 | +.09 | -.05 | +.1 | +.04 | +.04 |
| 5. Context Availability |  |  |  |  | - | -.09 | -.05 | +.18 | +.03 | +.02 | +.09 | -.13 | -0.04 | +.08 |
| 6. Valence |  |  |  |  |  | - | -.06 | -.61* | +.01 | -.06 | -.1 | +.1 | +.05 | -.11 |
| 7. Arousal |  |  |  |  |  |  | - | +.46* | +.13 | -.05 | +.18 | -.17 | -.11 | +.13 |
| 8. Pain-relatedness |  |  |  |  |  |  |  | - | -.03 | -.06 | -.06 | +.04 | +.04 | -.15 |
| 9. Zipf |  |  |  |  |  |  |  |  | - | -.04 | .00 | -.12 | +.35* | -.09 |
| 10. N |  |  |  |  |  |  |  |  |  | - | +.06 | -.17 | +.59* | -.51* |
| 11. OLD20 |  |  |  |  |  |  |  |  |  |  | - | +.71* | -.25 | +.29* |
| 12. Letters |  |  |  |  |  |  |  |  |  |  |  | - | +.01 | -.22 |
| 13. MaxFreqN |  |  |  |  |  |  |  |  |  |  |  |  | - | +.69* |
| 14. MeanFreqN |  |  |  |  |  |  |  |  |  |  |  |  |  | - |

*p < 0.0006

| Table 1C | Verbs | | | | | | | | | | | | | |
| --- | --- | --- | --- | --- | --- | --- | --- | --- | --- | --- | --- | --- | --- | --- |
|  | 1 | 2 | 3 | 4 | 5 | 6 | 7 | 8 | 9 | 10 | 11 | 12 | 13 | 14 |
| 1. Familiarity | - | -.49* | -.18 | -.05 | +.44* | +.2 | +.1 | -.01 | +.24 | -.3 | -.24 | -.01 | -.16 | +.19 |
| 2. Age of Acquisition |  | - | -.22 | +.05 | -.08 | +.15 | +.07 | +.19 | +.0 | -.04 | -.03 | -.11 | -.3 | +.31 |
| 3. Imageability |  |  | - | +.83* | +.39 | -.17 | +.05 | -.06 | -.14 | -.16 | -.21 | -.00 | -.16 | +.19 |
| 4. Concreteness |  |  |  | - | +.04 | +.3 | +.11 | +.09 | +.02 | +.00 | +.13 | +.08 | +.15 | -.15 |
| 5. Context Availability |  |  |  |  | - | +.03 | -.2 | +.13 | +.25 | +.23 | +.13 | -.08 | +.01 | -.07 |
| 6. Valence |  |  |  |  |  | - | -.05 | -.57* | -.18 | -.06 | -.16 | -.07 | -.01 | -.19 |
| 7. Arousal |  |  |  |  |  |  | - | +.57* | +.17 | +.16 | +.17 | +.26 | +.05 | +.03 |
| 8. Pain-relatedness |  |  |  |  |  |  |  | - | -.08 | -.15 | -.26 | -.22 | -.1 | -.13 |
| 9. Zipf |  |  |  |  |  |  |  |  | - | -.13 | -.41 | +.09 | -.01 | -.03 |
| 10. N |  |  |  |  |  |  |  |  |  | - | -.62* | -.06 | +.47* | -.48* |
| 11. OLD20 |  |  |  |  |  |  |  |  |  |  | - | +.22 | -.07 | -.14 |
| 12. Letters |  |  |  |  |  |  |  |  |  |  |  | - | -.21 | -.11 |
| 13. MaxFreqN |  |  |  |  |  |  |  |  |  |  |  |  | - | +.79* |
| 14. MeanFreqN |  |  |  |  |  |  |  |  |  |  |  |  |  | - |

*p < .0006

| Table 1D | Physical pain words | | | | | | | | | | | | | |
| --- | --- | --- | --- | --- | --- | --- | --- | --- | --- | --- | --- | --- | --- | --- |
|  | 1 | 2 | 3 | 4 | 5 | 6 | 7 | 8 | 9 | 10 | 11 | 12 | 13 | 14 |
| 1. Familiarity | - | -.37* | -.19* | -.22* | +.64* | +.11 | -.1 | +.06 | +.21* | -.02 | -.08 | +.15 | -.12 | +.21* |
| 2. Age of Acquisition |  | - | -.28* | +.13 | -.14 | -.01 | -.12 | -.23* | -.13 | -.07 | +.08 | -.03 | -.12 | +.16 |
| 3. Imageability |  |  | - | +.79* | +.23* | -.04 | +.17 | -.15 | -.0 | -.06 | +.16 | +.09 | -.11 | +.12 |
| 4. Concreteness |  |  |  | - | +.25* | +.11 | -.21* | +.26* | -.05 | +.05 | +.12 | -.08 | +.08 | -.07 |
| 5. Context Availability |  |  |  |  | - | -.04 | -01 | +.14 | +.1 | -.02 | +.09 | -.1 | +.03 | -.06 |
| 6. Valence |  |  |  |  |  | - | -.06 | -.6* | -.02 | +.02 | +.01 | -.02 | +.03 | -.07 |
| 7. Arousal |  |  |  |  |  |  | - | +.51* | +.11 | +.0 | -.14 | +.17 | -.12 | +.09 |
| 8. Pain-relatedness |  |  |  |  |  |  |  | - | -.05 | +.04 | -.01 | -.03 | +.03 | -.07 |
| 9. Zipf |  |  |  |  |  |  |  |  | - | +.02 | -.08 | -.02 | +.26* | -.07 |
| 10. N |  |  |  |  |  |  |  |  |  | - | +.04 | -.22* | +.5* | -.37* |
| 11. OLD20 |  |  |  |  |  |  |  |  |  |  | - | +.65* | -.31* | +.26* |
| 12. Letters |  |  |  |  |  |  |  |  |  |  |  | - | +.05 | -.18* |
| 13. MaxFreqN |  |  |  |  |  |  |  |  |  |  |  |  | - | +.7* |
| 14. MeanFreqN |  |  |  |  |  |  |  |  |  |  |  |  |  | - |

*p < .0006

| Table 1E | Social pain words | | | | | | | | | | | | | |
| --- | --- | --- | --- | --- | --- | --- | --- | --- | --- | --- | --- | --- | --- | --- |
|  | 1 | 2 | 3 | 4 | 5 | 6 | 7 | 8 | 9 | 10 | 11 | 12 | 13 | 14 |
| 1. Familiarity | - | -.25 | -.14 | -.04 | +.69* | +.12 | +.06 | -.02 | +.34 | +.04 | -.2 | +.21 | -.28 | +.16 |
| 2. Age of Acquisition |  | - | -.42 | +.1 | +.13 | -.35 | +.15 | -.23 | -.23 | +.01 | +.07 | -.14 | -.23 | +.18 |
| 3. Imageability |  |  | - | +.69* | +.29 | -.25 | +.01 | -.21 | -.0 | -.05 | -.07 | +.18 | -.04 | +.03 |
| 4. Concreteness |  |  |  | - | +.07 | +.35 | +.11 | +.28 | -.07 | -.07 | -.01 | -.1 | +.07 | -.1 |
| 5. Context Availability |  |  |  |  | - | -.06 | +.04 | +.07 | +.12 | -.05 | +.18 | -.13 | +.12 | +.04 |
| 6. Valence |  |  |  |  |  | - | -.2 | -.69* | -.26 | +.16 | +.21 | -.3 | -.11 | +.16 |
| 7. Arousal |  |  |  |  |  |  | - | +.14 | -.06 | +.03 | -.16 | -.02 | -.04 | -.03 |
| 8. Pain-relatedness |  |  |  |  |  |  |  | - | -.05 | -.04 | +.21 | -.22 | -.04 | +.02 |
| 9. Zipf |  |  |  |  |  |  |  |  | - | +.17 | -.0 | -.28 | -.07 | +.08 |
| 10. N |  |  |  |  |  |  |  |  |  | - | -.09 | +.0 | +.57* | -.48* |
| 11. OLD20 |  |  |  |  |  |  |  |  |  |  | - | +.61* | -.22 | +.04 |
| 12. Letters |  |  |  |  |  |  |  |  |  |  |  | - | -.11 | +.02 |
| 13. MaxFreqN |  |  |  |  |  |  |  |  |  |  |  |  |  | +.75* |
| 14. MeanFreqN |  |  |  |  |  |  |  |  |  |  |  |  |  | - |

*p < .0006

| Table 1F | Physical pain nouns | | | | | | | | | | | | | |
| --- | --- | --- | --- | --- | --- | --- | --- | --- | --- | --- | --- | --- | --- | --- |
|  | 1 | 2 | 3 | 4 | 5 | 6 | 7 | 8 | 9 | 10 | 11 | 12 | 13 | 14 |
| 1. Familiarity | - | -.33* | -.27 | -.15 | +.73* | -.05 | -.18 | -.02 | +.13 | -.06 | -.0 | +.06 | -.02 | +.17 |
| 2. Age of Acquisition |  | - | -.23 | -.04 | -.13 | -.03 | -.18 | +.2 | -.23 | -.08 | -.03 | +.12 | -.02 | +.05 |
| 3. Imageability |  |  | - | +.75* | +.26 | -.16 | +.13 | -.26 | +.06 | +.01 | -.21 | +.28 | -.02 | +.04 |
| 4. Concreteness |  |  |  | - | +.14 | +.25 | -.3 | +.37* | -.14 | -.05 | +.16 | -.26 | +.02 | +.04 |
| 5. Context Availability |  |  |  |  | - | +.1 | +.04 | +.18 | +.05 | +.04 | +.03 | -.01 | +.0 | -.12 |
| 6. Valence |  |  |  |  |  | - | +.01 | -.69* | +.03 | +.01 | -.02 | +.02 | +.05 | +.0 |
| 7. Arousal |  |  |  |  |  |  | - | +.47* | +.05 | -.03 | -.05 | +.04 | -.03 | +.01 |
| 8. Pain-relatedness |  |  |  |  |  |  |  | - | +.08 | +.06 | +.02 | +.01 | +.05 | +.03 |
| 9. Zipf |  |  |  |  |  |  |  |  | - | -.04 | -.31* | +.05 | +.03 | -.04 |
| 10. N |  |  |  |  |  |  |  |  |  | - | +.06 | -.3 | +.47* | -.37* |
| 11. OLD20 |  |  |  |  |  |  |  |  |  |  | - | +.69* | -.51* | +.27 |
| 12. Letters |  |  |  |  |  |  |  |  |  |  |  | - | +.26 | -.22 |
| 13. MaxFreqN |  |  |  |  |  |  |  |  |  |  |  |  | - | +.71* |
| 14. MeanFreqN |  |  |  |  |  |  |  |  |  |  |  |  |  | - |

*p < .0006
